# Notices

**Published:** 1866-09

**Authors:** 


					﻿NOTICES.
“New York Social Science Review;—a Quarterly Journal
of Sociology, Political Economy and Statistics, edited by Simon
Sterne and J. K. H. Wilcox, $4 a year. Nos. 1 and 2, for January
and April, 1866, bound in one cover, price $2 50. by mail. It
contains among other things, the “International Almanac for
1866,” which of itself is invaluable as a book of reference for
complete and latest and most authentic statistics geographical,
political, social and indd§trial; specially interesting now to all
intelligent men as it gives the population,' size, military and na-
val strength of all civilized countries; natures of their govern-
ments and constitutions; names, ages, qualifications, &c., of the
various rulers of the old world and the new ; size and population
of all our states, &c., &c., with a list of all the Banks, Stock-
Companies, Dividend values, &c.
The Southern Presbyterian Review, issued quarterly, $3 a
year, at Columbus, S. C., 100 pps, 8 vo., well executed on good
paper. The March Number contains Puritanism and Presbyte-
rianism ; St. Paul’s vision and natorv, by John H. Bocock, D. D.
of Va.; The Relation of State and Church, by Rev. R. S. Gledney,
of Miss.; Life and Times of Bertrand Du Guesclin, by Rev. A.
T. Dickson, of S. C.; Northern and Southern views of the Prom-
ise of the Church, by the Rev. Prof, J. B. Adger, D. I).
The Review is commended to the patronage of all good men
of liberal views in the whole Presbylerian Family. Would
it not be a good plan for all now that the State is able to
take care of herself, to join hands under the banner of a true
Presbyterianism as it existed in the days of the Revolution, and
make a resistless stand against sin and Satan, and under the
battle cry of “Forwarts Brudern,” place the standards of the
church in every town and village in the Union, the motto of
every company corps and division being, “Love One Another.”
Why Not ?—This book of 91 pages is sent post-paid in paper
binding, for 50 cents ; in neat cloth, $1.—The editor is the poor-
est hand in the world to go at things in a roundabout way. Ho
prefers to come right to the point. This book is published by
Lee *and Shepard, Boston, Mass. It is a Prize Essay by H. R.
Storer, M. D., of Boston, and is issued for general circulation by
order of the American Medical Association. And now for the point.
An increasingly large number of persons apply to city physici-
ans to destroy the child before its birth for one of three reasons—
io hide shame, to avoid the trouble of rearing children, or to
limit population. This book shows, authoritatively, the often
danger to life, and the infallibly serious effects on the constitution
in cases where the life of the mother is not lost. It is a public
humanity to publish such a book. It is needed, greatly needed
and should be seen by every husband and wife in process of a
family. Our book on “ Sleep,” (Si.50 by mail,) treats mainly on
this and kindred subjects; but Dr. Storer’s publication is more
scientific, treating on this one branch.
Messrs. Broughton & Wyman, Managers of the New York (13
Bible House) Branch of the American Tract Society, of 28
Cornhill, Boston, have just issued “ Pleasant Grove,” by Alice
A. Dodge, pp. 208., being sixteen narrations for children, well
adapted to foster “a more intense and firmer purpose” to prac-
tice what is true, lovely and good. “ Nellie Newton.” Pp. 144.
Showing, in a striking manner, the value of patience and perse-
verance as means of success in life, and the building up of char-
acters strong, useful and good. “ Lift a Little.” By Mrs. J. B.
Ballard. Pp. 80. Containing eight stories for little children,
teaching them to dare to do right always; why some are not
always happy; meaning to do good, lifting the old quilt of a
Christmas morning, to show all the beautiful things for the en-
couragement of good boys and girls.
Strong Tea.—A little girl, three years old, was left in a room
by herself in New York, and a few hours after drinking some
black tea from a cup, died. It is not uncommon for servants,
and even mistresses, to drink during the day the coffee and tea
left after the regular meals. It is a pernicious habit, leading to
nervousness, fretfulness, and general ill health, and is only sec-
ond, in its ill effects, to constant “tippling” among men, or the
frequent use of snuff and the tobacco pipe. Any habitual stim-
ulation, beyond that of the natural food and drink, tends always
to injure the health, weaken the mind, sour the disposition, and
shorten life.
Hot Weather.—In tiling a roof in 47th street, New York, in
July, 1866, the workmen found the thermometer to indicate a
heat of 137 degrees. About the same time a United States Mon-
itor in the torrid zone gave a heat in the engine fire room reach-
ing 150 degrees Fahrenheit, with the effect to induce spinal
disease, attended with violent convulsions, but none died.
Hydrophobia.—James Kirby, of Waterville, N. Y., aged 16
died on the 5th of July, 1866, in violent convulsions. About
three months before he had been bitten by a dog, yet no ill
effects had been experienced from the bite until the day before
he died. Dogs do not perspire except in the tongue. When they
become mad they froth at the mouth, showing that the perspir-
ing functions are changed. It was narrated, some years ago,
that a Frenchman, having been bitten by a dog, determined to
avoid hydrophobia by steaming himself to death ; but the effort
caused profuse perspiration, and he escaped hydrophobia, and
death also. In the light of these facts, a steam bath might be
tried in the case of a person suffering an actual attack of hydro-
phobic convulsions. Readers would do well to remember this,
and that a good steam bath may be extemporized by putting hot
stones under an open-seated chair, cover the patient with a blan-
ket, and pour water on the stones.
The Scientific American, which has every week valuable
suggestions in reference to domestic comfort and convenience,
besides its wealth of strictly scientific articles, says that ice may
be kept a surprisingly long time by stretching several inches of
cotton batting on a pasteboard, or a half dozen thicknesses of
newspaper, broader than the pitcher ; sew the longitudinal ends
together, so as to receive the pitcher ; let it stand on a cushion
of the same material, and put a pillow over the top. We had
ourselves tried a similar expedient two days before to preserve
some ice cream, and were giatified at the success of the experi-
ment. This is noticed for the benefit of the sick in localities
where ice can be obtained with difficulty, or from long distances.
—Or put in a vessel to be kept between two pillows, or hang the
ice in a well, just above the water—a twenty pound lump will
sometimes keep thus a day or two.
Car Riding.—One of our most respected physicians was riding
in a street car (Londoners call them “Tramways”) with his elbow
jutting out of the window ; the tongue of another vehicle came
in collision, and fractured the arm. This is the third accident of
the kind in New York city which has come to our notice. A
woman was almost burned to death recently, her clothing having
taki n fire from a spark from the locomotive while she was stand-
ing on the platform between tw’o cars.
The railroad between New York and Philadelphia, via New
Brunswick, has carried millions of passengers in the last twenty-
five years, and not a single life has been lost of any passenger
while in the cars. The practical inference is, that when you
ride in a vehicle keep your arms and head within, and avoid the
platforms of rail-cars, and the roofs of canal-boats.
.Epidemic Cholera, by J. S. Webster, M. D. Published by Mil-
ler, Wood & Co., 15 Laight street, New York: 48 pp., paper
cover, sold for 25 cents, is one of the most truthful and instruc-
tive issues for popular use that has lately come under our notice.
Its evident object is to get at the truth in theory and practice,
and abide by its teachings.
Our Daughters.—The New York Observer, of July 26th says:—
“ The Anniversary Exercises of the Misses Bucknall’s School,
recently of New York city, were held on the 13th inst., at New
Brunswick, N, J., a large audience being present. Prayer was
ffered by the Rev, Prof. Doolittle, after which the Senior Class,
with great credit to themselves and to the institution, engaged
in the critical analysis of the English language. The Composi-
tions of the Graduating Class, embracing the Valedictory, elicited
much commendation. After the distribution of the prizes, one of
the Principals delivered a parting address to the Graduates, pre-
senting them with their diplomas. Music was interspersed
through the exercises, which were closed with an eloquent ad-
dress by Kev. Dr. W. J. K. Taylor.”
Misses Laura Acker, Irene Birdsal (valedictarian), and Miss
Ellen H. Hall, each of whom received three or four prizes for
proficiency in several departments of study, were graduates from.
New York city.
Health and Disease.—Sent by mail for $1 60, shows that
there is no good health without a daily action of the bowels.
That to secure this by medicines or injections always leaves the
system in an unfortunate condition, and that the only natural,
safe and efficient method is the judicious adaptation of food, in
quality and quantity, to the need of each case. A book of such
great importance to human health and comfort, and of such uni-
versal application, written for popular use, has not been pub-
lished hitherto. The views are neither new nor original with us,
hence we praise the idea of the "book without praising ourself.
We want the people to understand, and we want it to be taught
to children as soon as they begin to be seven years old, that a
failure of the bowels to act once in every twenty-four hours, will
always and inevitably be followed by some symptom or actual,
and often fatal sickness in forty-eight hours ; and that a man
never gets well of any known disease until the bowels begin to
return to one action daily.
The great Cholera Symptom.—Many a reader of the follow-
ing sentence will die of cholera from inattention to it, that in
cholera times a forcible, painless, large, pale, thin weakening
discharge from the bowels, is Cholera begun ! and death will
follow in twenty-four hours unless it is attended to, the best
way of doing which is to lie down flat on a bed and stay there,
eating ice if thirsty, and keeping still and warm until a physician
can be had. (See January number of this Journal for 1866, sent
by mail for fifteen cents.)
In common diarrhoea the evacuations are yellowish and gri-
ping.
In cholera they are whitish and painless.
In dysentery they are scant and bloody, with distressing de-
sires, but inability to do anything but strain.
Bilious diarrhoea is a healthful process, and ought not to be
interfered with. The discharges are knot very thin, are always
black, green, or yellow, attended with a great deal of rumbling,
or darting, transient pains. For this, the best mode of procedure
is io be quiet, keep warm, eat nothing, take nothing, and send
for a physician.
If ! but what is the use of saying anything about it ? Men
will commit suicide, run the risk of dying any night, will sacri-
fice a good night’s rest, and ensure a weary waking in the morn-
ing, with a day of miserable fretfulness and nervousness follow-
ing, for the momentary gratification of their gormandizing throats,
rather than practice a little self-denial, and eat absolutely noth-
ing in warm weather, especially after a noon-day’s meal, but a
single piece of bread and butter, taking with it a single cup of
tea. If any man, woman or child, who reads this, will practice
it for a single week, and does not at the end of that time feel
better, sleep better, and have a more lively, cheerful temper, we
will send such the Journal ot Health, without charge, for 1866,
if the trial is made during this September, 1866. Strangers have
come to us, others have written to express their gratitude for the
suggestion of light suppers, in consequence of the greater happi-
ness of mind, and the greater bodily enjoyment following on the
trial.
Catarrh.—A gentleman from Canada West writes, July 26tb,
1866, to P. C. Godfrey of 823 Broadway, New York, “ Enclosed
please receive $20, for which express to me the value in your
catarrh remedy, I got some from you a short time ago for a
daughter, and it really has done her good,” This is inserted to
give us an opportunity of saying to our subscribers, we do not
prescribe for catarrh cases, it is not in our line of practice ; but
if you have catarrh, and are sure that it is catarrh, by the symp-
toms of more or less fulness in the parts connected with the
throat, and more fulness in head, watery nose and eyes, or ill
smell of the discharges from the nose, and if, in addition, you are
so unwise as to go to the advertising men of the large cities,
who will undertake to cure any case of catarrh for three hundred
dollars provided it is paid in advance, rather than cousult your
family physician—better risk five dollars in the trial of God-
frey’s remedy; for if it does you no good the money will be re-
turned, and “no questions asked and if good does follow its
use, then you have saved $295, a part of which, in gratitude, you
ought to expend in purchasing a full set of Hall’s Journal of
Health, 12 volumes bound in muslin, and two volumes Fireside
Monthly, now discontinued, bound uniformly with the Journal, for
$21, for reciprocity of good turns prumotes the general good.
OPEN FIREPLACES.
It is not possible to supply a pure warmth by any furnace
ever invented, unless it simply heats water or air, out of which
is given the caloric necessary to make a dwelling comfortable.
But warming houses by steam, hot water, or hot air, costs, for
an ordinary residence, about eight hundred dollars, which
makes it impracticable—places this luxury wholly beyond four ,
fifths of all the households in the land. That the heat which
comes from any furnace through an ordinary register, although
the coals are red-hot, is a sickening stench, can be demonstrated
any moment in a winter’s day; it is sending into a room an in-
cessant stream of air, almost wholly divested of its oxygen,
which is the element for which alone air is breathed at all; nor
is this all—the oxygen has not only been abstracted, but sul-
phurated hydrogen and carboneted hydrogen, which are
among the most noisome smells in nature—that of rotten eggs—
replace the oxygen; and that such an atmosphere, steaming into
our parlors, and dining-rooms, and chambers, can not be other-
wise than most pernicious to health, only but an idiot can deny.
Every year new patents are coming out, claiming to meet the
failures of their predecessors, proving conclusively that all pre-
vious ones have been signal and lamentable failures.
It may be a more potent and convincing argument against
the pestiferous effects of furnace heat, at least in the minds of
some, that it ruins the furniture and the woodwork of all
buildings into which it is introduced.
Open wood-fires, the most cheery and delightful of all modes
of house-warming, are too expensive, and are exceedingly
troublesome. The common open grates for coal are the next
best, but they fail to give a comfortable heat in the coldest
weather; they fail to keep the feet warm, which is the most
important part of the body to be kept agreeably heated; and, in
addition, the very instant the coal in the grate is touched, the
whole room is filled with a fine dust, which settles on the .paint-
ings, the furniture, the carpets, and the very clothing in the
drawers, making dingy the most polished surfaces, scratching
the furniture and the gilding, and grinding out the carpets by
the flinty dust.
But there is a method of warming houses, cheaper than
grates and more efficient, giving almost none of their dust;
incomparably less troublesome than wood-fires, while the heat
is just as genial and quite as pure; the fire needs replenishing
but once a day, never requires a poker, if properly attended to;
gives very little dust, keeps the feet warm, and keeps before
the eyes the cheery sight of a broad bed of burning, glowing
coals. In short, it is a plan for warming houses, which has
never, in all its points, been surpassed—has never been equaled.
It is Dixon’s low-down grate. It is believed that there is
scarcely a single educated physician in Philadelphia, who owns
the house he lives in, who is not supplied with one or more
of these delightful luxuries. They cost from twenty-five
dollars each and upward, and are placed in stead of an ordinary
fireplace or grate in the course of a few hours.
Three fourths of the heat of a grate or fireplace goes up the
chimney, and is wasted. Dixon’s Philadelphia low-down grate,
by a moderate extra expense, can be so arranged that all the
ashes are conveyed into the cellar, and the otherwise wasted
heat is saved to a considerable extent, and conveyed into the
rooms above; hot the heat of burning coals, but air is brought
from out-doors, carried behind the chimney-back, heated with-
out coming in contact with the coals, and is conveyed into the
room above by an ordinary register, not in a sulphurous odor,
but simply in the shape of pure air warmed, which is of ines-
timable value for sitting-rooms, chambers, and nurseries. We
had one of these admirable contrivances put in our house in
1859, and every additional year only increases our appreciation
of the luxury. This notice has been written without the know-
ledge of the manufacturer, and will surprise him as much as
any one of our readers; but it would add so much to the health
of families, both in town and country, whether they burn soft
coal, anthracite, or common wood, for it is adapted to the con-
sumption of any kind of solid fuel, that we feel constrained to
bring it thus prominently forward, and the more fearlessly
because we know whereof we affirm. To save us the expense,
time, and trouble of answering letters of inquiry, our readers
will please address T. W. Dixon, 1324 Chestnut street, Phila-
delphia, or his agents, Mead & Woodward, 37 Park Row, New-
York City
PACIFIC RAILROAD.
Such is the conformation of the earth’s surface and the relative
position of the countries of the world in reference to trade, com-
merce, and manufacture, that the great pathway of nations is des-
tined to be that which connects the Mississippi river with the
western shore of the Pacific Ocean, by rail.
As between China, Japan, Australia, and the East Indies, on the
one hand, and the United States, with England and Western
Europe, on the other, the Pacific railway would save months of
time in transit—and time is money—hence the road will be built.
Not one line, but two; for two are imperatively required. The
necessities of the times, martial, civil, social, and commercial, will
have them, and that too, in a much briefer space than most persons
imagine. One from Oregon to St. Anthony’s Falls; the other from
San Francisco by way of Texas to New Orleans. Then, by reason
of the trade winds going and returning across the Pacific, the
quickest route for travel or freight, from the Americas, will be from
New Orleans via El Passo, in Texas, to San Francisco; while from
China and adjacent countries, to the United States and England,
the most expeditious conveyance will be across the Pacific, and by
way of the line from the mouth of the Columbia or Puget Sound to
St. Anthony’s Falls, Minnesota, or the Mississippi river. A round
voyage thus, wall save between one and two months’ time; and that,
on a cargo worth millions of money, besides wages, is an item so
considerable, that private enterprise will greedily seize at the chance
of investment. __________________________
PIANO FORTES.
There is a time-honored and mammoth building cornering on
Fourteenth street and Third avenue, which has met the familiar gaze
of such of our citizens as have been accustomed to pass that way,
for perhaps a greater part of the present half century. This build-
ing stretching its immense length along two streets, is devoted exclu-
sively to the manufacture of the Worcester Piano, which has a name
for durability of structure and sweetness of tone which ought, if it has
not, to have' made the fortune of any man-of moderate ambitions.
But it is not as easy now as formerly, to make a fortune by strictly
honest dealing; if done at all, it is only until a man has become
decrepid and gray, and almost ready to take his departure on the
returnless journey; one of the reasons of this is found in an article
in the July number, headed “unskilled labor.”
Another, at least temporary drawback, as to a speedy fortune by
strict business integrity, is the want of means on the part of the
many, to secure the best materials for their particular handicrafts.
Sometimes on account of a want of foresight or thrift, or a still
more unpardonable want of knowledge, materials are needed for
the construction of a superior article, which no money can purchase,
and time only can procure the needed supply.
Too many of our mechanical men live from hand, to mouth, and
the material purchased yesterday, must be used to-day; in proof,
look at any floor in any brown stone or marbled front, in the whole
city of New York, constructed within the last five years, and it will
be scarcely possible to find a well-fitting door, an easy moving
drawer or window sash, while the joints in the floors will measure
from a quarter to half an inch or more. This is so undeniable, that
builders find it the shortest cut to say, that it is owing to furnace
heat; and yet, Forty-two Irving Place, which has a furnace only for
appearances, can show floors on either story half an inch apart at
the ends of the boards, and at the sides in proportion.
When, however, there is a business integrity, and abundant
means to employ the best materials in fabrics of any description,
two results always show themselves, a good name and an ultimate
prosperity; hence the reputation and success of the establishment in
question, whose instruments stand the test of all weathers, from
Canada to Cuba, and from the borders of the Atlantic to the shores
of the Pacific Sea.
To make this practically useful to all young mechanics, the secret
should be communicated, and it consists in three things:
1.	A faithful apprenticeship to a good master.
2.	A timely supply of the very best materials;
3.	Making them up without haste, and with the utmost careful-
ness.
In the case above, the wood of important parts is obtained years
beforehand; it undergoes a most minute examination as to its
soundness, passing through a long seasoning, according to the vary-
ing thickness and hardness of the particular wood, and if at the end
of this tedious process^ the material remains sound and hard, with-
out a blemish, it is used, and not otherwise. It is thus by making
each particular instrument as if for his own personal use, almost
living in the same building with the workmen, passing through
every room at any hour of the day, making the employes feel as
if they were watched every moment; it is by these means, we re-
peat, that the Piano Fortes of this house have acquired a reputation
at home and abroad, whidh requires an almost daily shipment to
other countries as well as to the various parts of our own.
To every young mechanic we therefore say, the path of a certain
and honorable success for you is,
1.	Be thorough masters of your calling; and,
2.	Give honest material and honest work to every article which
leaves your establishment.
Dr. R. T. Trall, whose Hydropathic establishment at 13 Laight Street,
New York, is so well known, has just published a book purporting to be
“ A Scientific and Popular Exposition of the Fundamental Problems in
Sociology, issued by William Wood and Co., of New York, and G. J.
Bums, Wellington Road, Chamberswell, London, England, sent post paid
for $2. 312 p„ 12mo., with Illustrations. Dr. Trall is a prolific writer,
and has published several books, which merit a very general circulation,
such as “Alcoholic Medication,” 30 cts.; “Women’s Dress,’’ 30 cts.;
“ Hygienic Cook-Book,’’ very valuable, 30 cts.; Rethes’ “ Manual of Gym-
nastics,’’ 40 cts. Turkish Baths at $1.50 each, where applicably adminis-
tered are of very great value ; they are artificial “ sweats,” and are often a
thousand times better than physic!
In the heat of summer it is especially desirable to preserve the entire
person as pure and clean as possible; the feet are particularly liable to
acquire an ill odor; they should be placed in water, warm is best,
ankle deep for a minute or two, then wiped dry and then wash face, hands,
armpits and lastly the feet with a mixture named in this Journal several
years ago ; two tablespoons of Hartshorn water, called “ Aqua Ammonia’*
by Druggists, in a basin of water ; or a tablespoon full or two of common
spirits of hartshorn in the same amount of water ; it not only removes all
odor, but leaves the skin most perfectly clean ; but put on a clean pair of
stockings every morning.
Brief Biographical Dictionary,by Rev. Charles Hole, of Trinity Col-
lege, Cambridge, England, with additions and corrections by Wm. A.
Wheeler, AM., assistant editor of Webster’s Dictionary and author of
Noted Names of Fiction, etc., published by Hurd and Houghton, 459 Broome
St., New York, 1866. 453 pp. 12mo Sent postpaid for $2.00 This book
gives, in alphabetical order, in one line, the most eminent names of the
dead, the year of their birth and death and four or five words descriptive
of the character of their lives; and this is human fame and human worth
and human life, to be compressed in a single line. Caesar C. Julius, Dicta-
tor, born 100 B.O. died 44 B.C.; Bonaparte, Napoleon I. Emperor, born
1769, died 1821. We eagerly looked for some of the revered names of our
own time. Archibald Alexander, American—Divine, born 1772, died
1851; James Waddell Alexander, D.D., (our own Pastor,) American Scholar
and Writer,born 1804, died 1859 ; Joseph Addison Alexander, D.D.,(Brother)
Divine and Linguist, born 1809, died 1860 ; Charles Caldwell, (our honored
medical preceptor,) Medical and Miscellaneous writer,born 1772, died 1853.
It is a most interesting and valuable book, destined to be in the library of
men of all professions, of poets, scholars, writers ; men of our time every-
where will make it a standard book of reference ; the American compiler
has added very greatly to the value of the English edition by adding very
many American names; the public will be glad to know that he is pre-
paring a similar work to embrace the names of the distinguished living.
				

